# Assessing digital health curriculum needs: a mixed-methods study of student and faculty perspectives in a Singapore medical school

**DOI:** 10.1080/10872981.2026.2726067

**Published:** 2026-09-01

**Authors:** Saumya Bajaj, Eng Chun David Goh, Yih Yng Ng, Zheng-Wei Lee

**Affiliations:** a Digital Innovation Office, Centre for Healthcare Innovation, NHG Health, Singapore, Singapore; b Lee Kong Chian School of Medicine, Nanyang Technological University, Singapore, Singapore

**Keywords:** Artificial intelligence in healthcare, digital health, electronic health records, mixed‑methods research, undergraduate medical education

## Abstract

**Background:**

The rapid advancement of digital technologies has transformed healthcare delivery, creating an imperative for medical education to integrate digital health (DH) competencies into undergraduate training. Despite growing recognition of its importance, progress in embedding DH literacy within formal medical curricula has been uneven and slow.

**Objective:**

This study examined DH education within an undergraduate medical programme by integrating faculty and student perspectives to identify curriculum development needs and inform evidence-based educational strategies.

**Design:**

A mixed-methods study was conducted at a Singapore medical school offering a 5-year undergraduate curriculum. Semi-structured interviews were conducted with four faculty members as DH content experts, exploring curriculum design, pedagogical strategies, and competency expectations. A 24-item survey assessing knowledge, attitudes, and usage of DH tools was administered to students across pre-clinical and clinical training, with 131 responses from 848 eligible participants. Qualitative data were analysed using grounded theory-based thematic analysis, whilst survey data were analysed using descriptive statistics.

**Results:**

Faculty interviews identified three key themes: curriculum gaps requiring more practical and clinically-oriented DH teaching, structural barriers including time constraints and rapid technological change, and complex stakeholder ecosystem influencing curriculum development. Student survey results revealed a persistent theory-practice gap across DH domains. Students reported high self-perceived knowledge in DH applications and artificial intelligence (AI) but consistently lower confidence in practical application, particularly in electronic health records (EHR) and telemedicine. Clinical students paradoxically rated their EHR knowledge lowest despite greater clinical exposure. Students expressed strong interest in programming, data analytics, and hands-on AI, favouring applied DH skills with direct clinical relevance.

**Conclusions:**

Medical curricula struggle to translate DH conceptual knowledge into practice, reconcile divergent competency expectations between students and faculty, and remain current amid rapid technological change. Tiered competency models, coupled with experiential learning approaches and continuous faculty development, represent a promising strategy for preparing future physicians for digitally enhanced healthcare delivery.

**Clinical trial number:**

Not applicable.

## Background

The rapid advancement of digital technologies has fundamentally transformed healthcare delivery, creating an imperative for medical education to evolve in parallel. Electronic health records, telemedicine platforms, artificial intelligence (AI)-driven diagnostic tools, and wearable health monitoring devices are now integral to routine clinical practice, influencing clinical workflows, decision‑making, and patient engagement [1]. As healthcare systems increasingly rely on digitally enhanced models of care, medical professionals are expected to possess not only traditional clinical competencies but also digital literacy skills for critical appraisal and effective use of these technologies in patient care.

Recognising this shift, medical education institutions and professional bodies worldwide have emphasised the importance of integrating digital health (DH) competencies into undergraduate training. The Association of American Medical Colleges (AAMC) has identified preparation for digitally enhanced healthcare as a core area of emerging physician competency [2], and frameworks have been developed to define DH competencies and capabilities for medical graduates, aiming to bridge the gap between evolving clinical practice and formal medical education [3,4].

Despite growing recognition of its importance, progress in embedding DH literacy within formal medical curricula has been uneven and slow. Across diverse geographic contexts, DH and AI education remains only partially embedded within undergraduate medical curricula, typically offered as pilot initiatives, electives, or student‑driven activities rather than as core curricular components [5]. This limited institutional incorporation persists despite consistently strong student interest and widespread support for mandatory DH training, highlighting a clear misalignment between learner expectations and curricular provision [6,7].

Empirical evidence highlights substantial regional gaps. In the United States, an analysis of 69 medical schools identified 103 innovation and technology‑related opportunities, most of which existed as electives, dual‑degree programmes, or extracurricular initiatives rather than required coursework [8]. This study cites DH as a component of the innovation and technology landscape, noting that 67% of surveyed student-led organisations included ‘digital health’ as a thematic focus, and contextualising the broader need for I&T education against the rapid growth of digitally driven healthcare—including video consultation platforms, wearable sensors, home testing kits, and the use of deep learning algorithms for diagnostics. European medical students report particularly pronounced deficiencies across a broad range of DH competencies (“eHealth skills”)—including clinical decision support systems, remote patient monitoring, AI applications, mHealth and telehealth—with over half rating their eHealth skills as poor or very poor despite strong agreement that additional DH education is needed [9]. These deficiencies have been attributed to multiple barriers operating at various levels, including the lack of coordinated formal education, insufficient faculty resources, an already high curricular workload, and the absence of accreditation of DH literacy within national medical education frameworks. Implementation challenges are further compounded in low‑ and middle‑income countries, where infrastructural and resource limitations constrain curricular reform [10].

In Singapore, national initiatives such as the Smart Nation programme [11] and the implementation of a National Electronic Health Record system [12] underscore the centrality of DH to healthcare delivery. At the institutional level, public hospitals such as Tan Tock Seng Hospital and the National University Hospital utilise Epic, a comprehensive Electronic Health Record (EHR) platform that supports clinical documentation, order entry, results review, and care coordination across inpatient and outpatient settings. Epic also forms the technological foundation of the National Electronic Health Record (NEHR) system, a centralised repository that aggregates patient health information across different healthcare providers, enabling care continuity and longitudinal tracking of patient health data across the public and private sectors. At the patient-facing level, HealthHub provides citizens with direct access to their medical records, laboratory results, appointments, and health metrics across public healthcare institutions. Together, these platforms represent the broader ecosystem of DH tools that medical graduates are increasingly expected to navigate competently in clinical practice. Although all three medical schools in Singapore have introduced DH–related content, approaches remain institution‑specific and non‑standardised. The differing emphases by different medical schools—team‑based learning at Lee Kong Chian School of Medicine, research‑oriented training at Duke‑NUS, and innovation‑focused education at NUS Yong Loo Lin School of Medicine—have not been systematically leveraged for coordinated national digital competency development [13]. While prior studies have explored stakeholder perceptions and identified DH knowledge gaps, there remains limited empirical research examining how undergraduate medical curricula are adapting in practice to prepare students for a digitally enhanced healthcare environment.

Addressing these challenges requires a nuanced understanding of both educator perspectives on curriculum design and implementation, and students’ self‑perceived competencies, attitudes, and learning needs. However, studies that integrate these perspectives within a single institutional context remain scarce. In particular, there is a need to examine how faculty capacity, curricular constraints, and institutional resources interact with student expectations and preparedness to shape DH education. To address this gap, the present study adopts a convergent mixed‑methods approach to examine DH education within an undergraduate medical programme, aiming to identify faculty‑perceived challenges in curriculum integration and to evaluate medical students’ perceptions of their DH competencies, attitudes, and learning needs. By triangulating educator and learner perspectives, this study provides a preliminary, context-specific needs assessment that seeks to inform evidence‑based curriculum development strategies that are responsive to the demands of rapidly evolving digital healthcare.

## Methods

### Research context

This study was conducted in a Singapore undergraduate medical school offering a newly reformed 5-year MBBS (Bachelor of Medicine and Bachelor of Surgery) curriculum. The first two years comprise predominantly campus-based learning focused on foundational biomedical sciences, clinical skills, and professional development. The subsequent three years consist of clinical placements across affiliated healthcare institutions, where students undertake workplace-based learning and clinical rotations in authentic healthcare settings. As Singapore continues to advance its national digital transformation agenda, DH has become an increasingly important component of healthcare delivery, with technology and AI tools playing growing roles in clinical decision-making and patient care. This study sought to examine current educational practices and identify opportunities to strengthen DH education within the undergraduate medical curriculum.

### Faculty interviews

Four faculty members with content expertise in the DH curriculum were interviewed via secure video conferencing (i.e. Zoom) using a semi-structured interview guide. These participants represented key content experts involved in the design and delivery of the DH curriculum, including Scientific and Clinical Leads, clinician-scientists, and data analysts. Given the focused study aim and the participants' specialised expertise, the interviews were considered to provide sufficient information power [14] to explore curriculum needs, implementation challenges, and competency expectations in DH education.

The interview guide developed for this study was based on review of relevant literature and theoretical frameworks [4,15], with additional refinement through input from the research team. Interview questions explored various aspects of incorporating DH into medical education, including faculty roles, competency expectations, curriculum design, pedagogical strategies, and challenges (Appendix 1). Participants were recruited via email invitation to participate in video conference interviews. Acceptance of the invitation was taken as written consent to participate in the study. At the commencement of each interview session, explicit verbal consent was also obtained from each participant prior to proceeding. Each interview lasted between 30 and 60 minutes. All sessions were audio-recorded with participant consent and subsequently transcribed verbatim using Microsoft Word's transcription function for qualitative analysis.

### Student survey

The student survey instrument was developed through a review of DH education literature and relevant theoretical frameworks [9,16]. These sources informed the scope and content of survey items, spanning domains of technology adoption and usage, eHealth and AI literacy, attitudes toward DH tools, and readiness for DH education and training. The final questionnaire comprised 24 Likert-scale items assessing students’ knowledge, attitudes, and usage of DH tools, with responses recorded on a five-point scale ranging from 1 (strongly disagree) to 5 (strongly agree), as well as three open-ended questions to capture qualitative insights (Appendix 2). Items were iteratively refined by the research team, and face validity was established through pilot testing with a student sample (*n* = 10) to ensure clarity, coherence, and interpretability of all items prior to full administration. The questionnaire was designed as an exploratory tool to map the breadth of students’ knowledge, attitudes, and usage across multiple DH domains, rather than as a psychometrically validated scale measuring a single latent construct. Quantitative data were collected using an online survey administered to students across pre-clinical and clinical years. Although clinical training formally commences in Year 3 (Y3), as the survey was administered at the start of the academic year, Y3 students were classified as pre-clinical given their limited exposure to clinical training at the time of data collection. Recruitment was voluntary and facilitated through cohort representatives - a designated point of contact for each year of study (Years 1 to 5) - via instant messaging channels. The survey, hosted on Microsoft Forms, remained open for two weeks (Aug - Sep 2024) and was accessible through a QR code link. Written informed consent was embedded within the survey platform, and all responses were anonymous with no personal identifiers collected. Demographic data captured included year of study and gender. Of the 848 medical students in the school's 5-year MBBS program, 131 responded to the survey.

### Data analysis

Survey data were analysed using descriptive statistics and visualised using Microsoft Excel. Inferential statistics were evaluated using a two-tailed, two-sample t-test. A *p*-value less than 0.05 was regarded as statistically significant. Qualitative data from interviews were analysed using grounded theory–based open coding and thematic analysis. Two researchers independently coded the transcripts, with discrepancies resolved through discussion to ensure consistency. Analysis focused on eliciting expert perspectives thus coding continued until thematic sufficiency was achieved, and data were organised into overarching themes. Both the quantitative and qualitative data were collected in parallel, analysed separately and integrated during interpretation.

### Ethical approval

Ethical approval for this study was obtained from the Nanyang Technological University Institutional Review Board (IRB-2024-839), and all procedures complied with the Declaration of Helsinki. All participants provided informed consent prior to participation, and confidentiality was maintained throughout. No identifiable information was collected, and all data were securely stored in accordance with institutional guidelines.

## Results

### A. Faculty perspectives on digital health education

Semi-structured interviews with DH content experts explored faculty perspectives on the current DH landscape. Analysis identified three interrelated themes influencing DH education: curriculum gaps, structural barriers, and the stakeholder ecosystem.

Faculty highlighted clear *curricular gaps*, particularly the need for a more practical and integrated approach to DH teaching. Participants emphasised that DH content should be more clinically oriented, “*We decided to make sure that the curriculum had a much more practical and much more medical oriented…*”. Participants highlighted the importance of integration across the curriculum: “*A curriculum which is more, perhaps more integrated.*” In addition, “*foundation and background of data, science and AI… understand what data is, how it can be used, stored, managed…*” was viewed as essential knowledge for enabling future doctors to critically appraise and appropriately apply digital technologies rather than using them as “black boxes.” Faculty also identified several ethical considerations that should form part of DH competency development. These included fairness and equity in the design and implementation of AI systems, with participants highlighting the importance of ensuring that "*there needs to be ethical considerations of the system that you built it must be fair to all the individuals which you apply the system to.*" Ethical competence was also linked to legal and regulatory awareness, as future clinicians were expected to understand that "*MOH has a regulatory frameworks for the use of AI in medicine*" and that they "*need to be aware of all these legal frameworks*." In addition, transparency was identified as an important consideration, with one participant noting that "*there are also other considerations like, you know, transparencies.*" Collectively, these findings suggest that faculty view ethical competency in DH as encompassing fairness, regulatory and legal governance, and transparency.

Despite recognising the importance of DH education, faculty identified significant *structural barriers* that limit curricular expansion. The most frequently cited challenge was time constraint within an already crowded curriculum. One participant stated, “*Other challenges would be the amount of time we have with the students. So the curriculum, as it is, is very tight*,” while another echoed, “*The challenge is always the time component… to fit more stuff.*” within the very limited curriculum space.

Another major barrier related to the rapid pace of technological change and the difficulty of keeping curricular content current. Faculty described, “*Given the pace at which the area is changing… the challenges of keeping it up to date.*” This challenge was further illustrated by the remark, “*Then suddenly there’s this cutting-edge stuff (that you want to teach)… but it goes by by the time I teach it…*”, reflecting how educational content often evolves more slowly than advances in DH technology.

Faculty described DH curriculum development as embedded within a broader *stakeholder ecosystem* encompassing students, alumni, clinical partners, and national regulatory structures. Participants noted substantial variation in student interest and engagement, with one faculty member observing, “*We have had students who are extremely interested… others who are less… this is just something that I need to learn.*” At the same time, student and alumni feedback was viewed as an important driver of curricular change, as reflected in comments such as “*The student feedback that has been used to drive changes in the curriculum*,” and “*We did reach out to existing students as well as alumni on what they perceive.*” Clinical partners were also identified as essential stakeholders in ensuring real-world relevance, with faculty emphasising the need to “*leverage quite a bit on our clinical partners… feedback from our clinical partner so that we can incorporate these concerns into the curriculum.*” Finally, alignment with national curriculum frameworks was described as a guiding influence.

### B. Students’ survey on DH domains

A total of 131 medical students completed the survey, comprising 76 males (58.0%) and 55 females (42.0%) (Table 1). Most respondents were from the clinical years (*N* = 98, 74.8%), with Year 4 students representing the largest subgroup (*n* = 87, 66.4%). Pre-clinical respondents accounted for 25.2% of the sample (*N* = 33), with female students constituting the majority across all pre-clinical years.

**Table 1.** Distribution of survey responses by year of study and gender.

	Gender		
Year (Y)	Male	Female	Total
***Pre-clinical year (N = 33)***			
Y1	3	8	11
Y2	3	5	8
Y3	4	10	14
***Clinical year (N = 98)***			
Y4	60	27	87
Y5	6	5	11
	**76**	**55**	**131**

#### B1. Digital health applications

In the domain of DH applications (eHealth services such as mobile health apps and DH resources, and telemedicine), both pre-clinical and clinical students reported high levels of self-perceived knowledge (pre-clinical 3.82 and clinical 3.93 on a 5-point scale) on e-health services and patient education (Table 2). They generally reported strong attitudes toward the relevance of digital tools for medical practice (pre-clinical 3.86 and clinical 3.70). Although differences were not statistically significant, clinical students demonstrated higher reported usage of DH applications overall (pre-clinical 2.93 vs clinical 3.18), with particularly elevated engagement in eHealth services (pre-clinical 3.91 vs clinical 4.23), likely reflecting greater exposure during clinical placements. Despite this, telemedicine usage remained notably low across both groups (pre-clinical 1.96 and clinical 2.13), highlighting a substantial gap in experiential learning. The consistently low telemedicine scores suggest that structured and deliberate opportunities for hands-on telemedicine practice, through simulation training or virtual ward experiences, are currently lacking in the curriculum and may be needed to better prepare students for emerging models of digitally enhanced care.

**Table 2.** Digital health applications: Knowledge, attitudes, and usage scores by training stage.

Digital health applications	Pre-clinical years, Y1-Y3 (*N* = 33)		Clinical years, Y4&Y5 (*N* = 98)	
	n	Ave. score	n	Ave. score
		**3.62**		**3.35**
***Knowledge***		***3.82***		***3.93***
eHealth services	33	3.79	97	4.11
Patient education	27	3.85	98	3.76
***Attitudes***		***3.86***		***3.70***
Workforce preparedness	32	3.88	98	3.69
Learning	32	3.72	98	3.76
Hands-on experience	31	3.74	98	3.42
Health disparities	33	4.12	98	3.93
***Usage***		***2.93***		***3.18***
eHealth services	33	3.91	97	4.23
Telemedicine	25	1.96	93	2.13

#### B2. Artificial intelligence (AI)

In the domain of AI, both pre‑clinical and clinical students reported comparable levels of self‑perceived knowledge (pre-clinical 3.76 and clinical 3.66) and similar frequencies of AI usage (pre-clinical 3.36 and clinical 3.60), indicating that foundational awareness of AI tools is relatively consistent across training stages (Table 3). However, clinical students demonstrated a statistically significant decline in knowledge regarding the practical application of AI in clinical settings (pre-clinical 3.76 vs clinical 3.12, *P* < 0.05). This decline in clinical years may reflect increased self-awareness among clinical students who, through practical exposure, have developed a more realistic assessment of the complexity involved in applying AI tools in clinical practice. Attitudinal scores revealed a pronounced drop among clinical students (pre-clinical 3.06 vs clinical 2.50), with the sharpest decline observed in items related to the anticipated impact of AI on professional roles and autonomy (*P* < 0.05). This attitudinal decline may reflect pre-clinical students' greater technological optimism, comfort with digital innovation, and idealistic expectations of seamless AI integration, whilst clinical students develop more nuanced perspectives informed by practical healthcare experience. This pattern may indicate growing scepticism or concern about how AI may alter the responsibilities and identity of future physicians. Taken together, these findings suggest that while students possess basic AI literacy, there is a clear need for curricular approaches that pair technical understanding with critical engagement on real-life application. Integrating structured practice opportunities alongside guided discussions on autonomy, bias, and role redefinition may help support more balanced and informed attitudes toward AI in clinical care.

**Table 3.** Artificial Intelligence: Knowledge, attitudes, and usage scores by training stage.

Artificial Intelligence (AI)	Pre-clinical years, Y1-Y3 (*N* = 33)		Clinical years, Y4&Y5 (*N* = 98)	
	n	Ave. score	n	Ave. score
		**3.45**		**3.63**
***Knowledge***		***3.76***		***3.66***
Concepts	33	3.58	98	3.73
Practical applications^*^	33	3.76	97	3.12
Practical challenges	33	3.82	98	3.66
Data	33	3.91	98	4.07
Ethics	33	3.73	98	3.68
***Attitudes***		***3.06***		***2.50***
Change in roles*	33	3.88	98	2.92
Training sufficiency	33	2.39	98	2.29
Autonomy*	33	2.91	98	2.31
***Usage***		***3.36***		***3.60***
Productivity	33	3.67	98	3.65
Learning	33	3.36	98	3.66
New tasks	33	3.30	98	3.60
Evaluation	33	3.12	95	3.46

^*^ Indicates a statistically significant difference (P < 0.05) in average scores by students in pre-clinical and clinical years.

#### B3. Electronic health records (EHR)

The electronic health records domain yielded the lowest scores across all three areas assessed and demonstrated a marked decline from pre-clinical to clinical years (pre-clinical 3.07 vs clinical 2.74) (Table 4). Clinical students reported particularly low levels of EHR knowledge (pre-clinical 3.80 vs clinical 2.02, *P* < 0.05), despite having substantially more exposure to EHR systems during clinical postings, again potentially indicating increased self-awareness. Both groups also rated their EHR usage poorly (pre-clinical 2.74 and clinical 2.86), with similarly low scores for learning and navigation skills, indicating insufficient hands-on training. These findings suggest that EHR competency represents a critical gap within the current curriculum.

**Table 4.** Electronic health records: Knowledge, attitudes, and usage scores by training stage.

Electronic health records (EHR)	Pre-clinical years, Y1-Y3 (*N* = 33)		Clinical years, Y4&Y5 (*N* = 98)	
	n	Ave. score	n	Ave. score
		**3.07**		**2.74**
***Knowledge***		***3.80***		***2.02***
Policies^*^	30	3.80	98	2.02
***Attitudes***		***3.00***		***3.22***
Accessibility	23	3.00	97	3.22
***Usage***		***2.74***		***2.86***
Navigation	22	2.82	98	3.01
Learning	23	2.65	98	2.71

^*^ Indicates a statistically significant difference (*P* < 0.05) in average scores by students in pre-clinical and clinical years.

#### B4. Student disposition toward inclusion of DH topics

Students expressed a generally positive and practice‑oriented disposition toward the inclusion of selected DH topics in the curriculum. The strongest interest was observed for programming and basic coding (*n* = 23), particularly when linked to concrete applications such as research data processing. Interest in AI usage (*n* = 15) such as prompt engineering and understanding the limitations of large language models (LLMs) reflected growing awareness of AI as a clinical support tool, with students emphasising its role in augmenting, rather than replacing, clinical decision‑making. Students additionally highlighted the need for hands‑on experience with AI tools in clinical settings, reinforcing a preference for experiential learning. Data analytics (*n* = 8) was also viewed favourably for its perceived utility in enhancing research capability and productivity. Finally, EHR training (*n* = 1) was identified as a necessary practical competency, particularly operational knowledge of hospital electronic medical record systems. Overall, students favoured applied DH skills with direct relevance to learning, research, and clinical practice.

## Discussion

This study provides a nuanced examination of DH education within an undergraduate medical curriculum by integrating student‑reported competencies with faculty perspectives on curriculum design and implementation. Overall, there was strong convergence between students and faculty regarding the importance of DH and the need for clinically relevant, applied learning approaches. Both datasets supported a foundational, scaffolded model of curriculum design rather than deep technical specialisation. In relation to student readiness, students reported variability in confidence which aligned with faculty perceptions of limited baseline knowledge, thereby highlighting the need for structured onboarding. Divergences emerged around perceived barriers, with students focusing on personal relevance while faculty emphasised institutional and curricular constraints. Together, the findings reveal three interconnected challenges that are increasingly prominent within medical education. Notably, there is a marked transition in medical students’ perspectives on DH, evolving from theoretical optimism during the pre-clinical phase to informed realism throughout clinical training. This shift underscores a persistent gap between theory and practice in DH education. Addressing this discrepancy necessitates reconciling the differing expectations of students and faculty. To this end, we propose a tiered model of DH competency, aimed at preserving curricular relevance and ensuring comprehensive development of DH skills amidst ongoing technological advancements.

### From theoretical optimism to informed realism in DH competency

The counterintuitive finding that clinical students reported lower EHR and AI knowledge despite greater exposure likely reflects the evolution from theoretical confidence to informed self-awareness [17]. Pre‑clinical students reported higher self‑perceived competency and more positive attitudes toward DH, likely reflecting early exposure to conceptual knowledge and aspirational narratives surrounding digital innovation [9]. This optimism may be reinforced by generational familiarity with emerging technologies and idealised expectations of how AI will enhance clinical practice [18].

However, as students progressed into clinical postings, confidence declined, particularly in domains requiring practical application such as electronic health records, telemedicine, and AI. Clinical immersion appears to expose students to the operational, ethical, and organisational constraints of digitally enhanced care. It prompts more critical self‑assessment as learners encounter real‑world workflow limitations, system inefficiencies, and professional accountability [19]. Overall, the results suggest a potential shift in medical students’ perceptions of DH competencies, characterised by a shift from theoretical optimism in the pre-clinical years to informed realism during clinical training. Similar developmental patterns have been observed internationally, where early enthusiasm for DH is tempered by real-world clinical experience that reveals gaps between theoretical preparation and practice demands [9,20]. This recalibration suggests that attitudes toward DH—and AI in particular—are shaped not only by knowledge acquisition but also by experiential exposure and the ongoing formation of professional identity [21,22], supporting the view that competence development in DH is inherently developmental and context-dependent.

### Persistent theory–practice gap in DH education

Importantly, the observed theory–practice gap in DH education appears to be structural in nature, rather than a deficit attributable to individual learners. Current curricula commonly introduce DH concepts during the pre-clinical phase through classroom-based teaching, while substantive experiential learning is deferred to clinical environments where instruction is often informal and inconsistent. This misalignment was particularly evident among clinical-year students, who paradoxically rated their EHR knowledge lowest despite greater exposure during clinical postings. Faculty interviews reinforced this finding, attributing the gap to limited curriculum time, crowded syllabi, and an over-reliance on ad-hoc learning opportunities within clinical settings.

Allowing students to encounter the operational realities of digitally enhanced care only after entering clinical postings risks further widening the divide between conceptual knowledge and practical competence. This pattern aligns closely with international literature describing DH education as conceptually framed but operationally underdeveloped. European medical students similarly report insufficient hands-on exposure to eHealth and EHR systems despite recognising their centrality to contemporary practice [9]. Scoping reviews further demonstrate that DH teaching is frequently confined to lectures or isolated modules, with limited opportunities for structured experiential learning [5,20]. The implications of this gap extend beyond educational satisfaction to broader concerns related to patient safety and clinical efficiency. Inadequate EHR training has been associated with documentation errors, workflow inefficiencies, and increased cognitive burden among novice clinicians [23,24]. Likewise, limited exposure to telemedicine during training may leave graduates underprepared for increasingly prevalent models of remote and hybrid care, further underscoring the need for earlier and more deliberate integration of experiential DH learning within medical curricula.

Addressing the theory–practice gap requires more than expanding DH content; rather, it necessitates a reorientation toward experiential, longitudinal learning. Simulation‑based EHR training, structured telemedicine role‑play, and supervised exposure to AI‑enabled tools have been shown to improve confidence and functional competence when integrated early and reinforced throughout training [25,26]. Embedding such approaches within core clinical teaching, rather than relegating them to optional or late‑stage activities, may be essential for closing this gap.

### Reconciling student and faculty expectations on DH competency development

Beyond the persistent theory–practice gap, this study highlights a critical need to reconcile student enthusiasm for emerging DH competencies with faculty uncertainty regarding appropriate expectations and curricular feasibility. Survey free‑text responses revealed strong student interest in advanced digital skills, including programming, prompt engineering, and data analytics, reflecting a desire to engage meaningfully with rapidly evolving technologies. However, this enthusiasm was frequently tempered by concerns about curricular burden, suggesting that students are simultaneously aware of the constraints imposed by an already dense medical curriculum.

Faculty perspectives echoed this ambivalence. While educators recognised the growing importance of DH, they expressed difficulty defining how much technical expertise is appropriate for generalist physicians and how such content could be accommodated within finite curriculum time. This uncertainty was compounded by the pace of technological change and challenges in maintaining up‑to‑date teaching expertise.

This divergence reflects a broader and well-documented debate within medical education regarding the scope and depth of DH training. Studies across multiple contexts report that while students value foundational digital literacy, they remain divided on whether advanced technical skills should be mandatory components of undergraduate education [7,27]. Faculty, in turn, often struggle to reconcile expanding competency expectations with constrained curricular space. In response, recent scholarship has increasingly advocated for tiered or scaffolded competency models, which differentiate core competencies required of all physicians from advanced skills aligned with specific interests or career trajectories [4,28].

### Continuous curriculum adaptation enabled by tiered models

Tiered models offer a pragmatic solution to the divergent expectations observed in this study. Core competencies—such as understanding data flows, interpreting AI outputs, recognising algorithmic bias, and applying ethical reasoning—can be embedded longitudinally across the curriculum to ensure baseline digital readiness. Advanced competencies, including coding or analytics, can be offered through electives, tracks, or ungraded modules, allowing motivated students to develop deeper expertise without overburdening the entire cohort. This approach aligns with WHO and DECODE frameworks, which emphasise universal digital literacy while acknowledging the need for specialised expertise in certain contexts [4].

Moreover, tiered competency models play a critical role in enabling continuous adaptation. Faculty consistently identified slow curriculum update cycles and limited DH expertise as barriers to integrating emerging technologies [13,29]. Students similarly reported gaps in AI ethics and real‑world application, highlighting the limitations of static curricula in fast‑evolving domains. By maintaining relatively stable core competencies while allowing advanced tiers to evolve rapidly, curricula can remain responsive without requiring wholesale redesign. Such modularity also supports targeted faculty development, enabling educators to upskill incrementally within defined competency domains. Faculty development programmes focused on digital literacy, pedagogy, and ethics are essential complements to curricular reform, as educators play a central role in modelling critical engagement with digital tools.

### Limitations

This study is strengthened by its convergent mixed‑methods design, which triangulates student and faculty perspectives to provide a comprehensive view of DH education. However, it is limited by its single‑institution context and reliance on self‑reported data, which may not fully capture actual competence. Students with pre-existing interest in DH may have been more responsive to survey and overrepresented. Consequently, response bias cannot be excluded. Findings should therefore be interpreted as reflecting engaged student perspectives and indicative trends, rather than as estimates of the entire population. Future research should explore longitudinal outcomes and multi‑institutional comparisons to further validate these findings.

## Conclusions

Taken together, this study demonstrates that while medical students recognise the importance of DH, medical curricula struggle to translate conceptual knowledge into practice, reconcile divergent competency expectations, and remain current amid rapid technological change. Tiered competency models, coupled with experiential learning and continuous faculty development, represent a promising approach to addressing these challenges and preparing future physicians for digitally enhanced care.

## Data Availability

The data collected and analyzed in the current study are available in an anonymized format from the corresponding author upon reasonable request and subject to institutional approval.

## Appendix 1

**Semi-structured Interview Questions for Faculty Interviews**

*Faculty role and teaching experience in digital health*

- What is your role in the digital health curriculum?
- What has been your experience in teaching digital health and AI technologies to medical students?

*Perceptions of student competence and competency expectations*

- What is your perception of students’ digital health and AI literacy?
- What competencies should students master in the digital health domain?

*Curriculum design and assessment*

- How is the curriculum structured across the five years?
- How do you choose what to teach in digital health and AI?
- How is digital health assessed (e.g. through in‑class quizzes, summative exams)?

*Curriculum effectiveness, challenges, and adaptation*

- What are the successes and challenges in teaching digital health?
- What feedback have you received from students?
- What are the challenges in keeping the curriculum up to date?
- How do you balance foundational knowledge with cutting‑edge advancements?

## Appendix 2

**Survey Questions—5-point Likert-scale Items**

*Digital health applications*

- I am familiar with eHealth services (e.g. HealthHub/Healthbuddy/NHG Cares/OneNUHS, etc.)
- Digital health resources that are primarily for patient education (e.g. on public hospital websites) help me understand the patient journey better
- Digital health training by the school is essential to prepare me for the workforce
- I am interested in learning about the application of digital health tools (e.g. medtech, entrepreneurship and innovation)
- I am interested in gaining hands-on AI/Digital Health experience through hands-on healthtech project work or a posting to a healthtech unit
- I believe that the increased use of eHealth services will increase health disparities for the elderly
- I have eHealth services (e.g. HealthHub/Healthbuddy/NHG Cares/OneNUHS, etc.) downloaded on my phone and use it
- I find it harder to take a history from a patient over telemedicine as opposed to in person

*Artificial Intelligence (AI)*

- I have a basic understanding of artificial intelligence (AI) concepts and technologies
- I am knowledgeable about how AI can be applied in healthcare.
- I am aware of the challenges in applying AI in clinical/patient care.
- I understand how data quality impacts on AI
- I am familiar with ethical issues in AI, such as bias, privacy, lack of transparency and explainability
- I believe AI will drastically change my role in healthcare in 5 years
- I am worried the training I am being provided does not equip me to cope with the use of AI in healthcare
- I am concerned that AI will reduce my autonomy to make clinical decisions
- I use AI tools to help me increase my productivity
- I use AI tools to learn new things
- AI tools have helped me perform tasks that I have not done before (draw pictures, write code, solve a complex problem etc.)
- I can evaluate AI applications for misinformation and inaccuracies

*Electronic Health Records (EHR)*

- I know how to comply to the policies and regulations of using electronic health records
- The data protection policies are very restrictive, limiting my ability to find patients to clerk
- I find it challenging to navigate through the digital patient medical records
- I find the learning curve to use the electronic health record system to be steep

*Open-text questions*

- What is the most interesting thing you have done using AI/Digital Health?
- Are there any design skills involving AI and Digital Health (e.g. Design Thinking, Agile, Lean UX, UI design) that you wish were part of the curriculum? If so, let us know and why.
- Are there any technical skills involving AI and Digital Health (e.g. programming, prompt engineering, analytics etc.) that you wish were part of the curriculum? If so, let us know and why.
